# Emergence and Evolution of Cooperation Under Resource Pressure

**DOI:** 10.1038/srep45574

**Published:** 2017-03-31

**Authors:** María Pereda, Débora Zurro, José I. Santos, Ivan Briz i Godino, Myrian Álvarez, Jorge Caro, José M. Galán

**Affiliations:** 1 Grupo Interdisciplinar de Sistemas Complejos (GISC), Departamento de Matemáticas, Universidad Carlos III de Madrid, 28911 Leganés, Madrid, Spain; 2 CaSEs - Complexity and Socio-Ecological Dynamics Research Group, Barcelona, Spain; 3 Department of Archaeology and Anthropology-IMF, CSIC (Spanish National Research Council), Barcelona, Spain; 4 Department of Humanities, Universitat Pompeu Fabra (UPF), Barcelona, Spain; 5 INSISOC. Departamento de Ingeniería Civil, Universidad de Burgos, Edif. “La Milanera”, C/Villadiego, s/n.09001, Burgos, Spain; 6 CONICET-CADIC. B, Houssay, 200, Ushuaia, 9410, Argentina; 7 ICSE-UNTDF, Los Ñires, 2382, Ushuaia, 9410, Argentina; 8 Department of Archaeology, University of York, The King’s Manor, Y01 7EP, York, UK; 9 GSADI. Department of Sociology, Autonomous University of Barcelona (UAB), Barcelona, Spain

## Abstract

We study the influence that resource availability has on cooperation in the context of hunter-gatherer societies. This paper proposes a model based on archaeological and ethnographic research on resource stress episodes, which exposes three different cooperative regimes according to the relationship between resource availability in the environment and population size. The most interesting regime represents moderate survival stress in which individuals coordinate in an evolutionary way to increase the probabilities of survival and reduce the risk of failing to meet the minimum needs for survival. Populations self-organise in an indirect reciprocity system in which the norm that emerges is to share the part of the resource that is not strictly necessary for survival, thereby collectively lowering the chances of starving. Our findings shed further light on the emergence and evolution of cooperation in hunter-gatherer societies.

Cooperation is critical for understanding how human societies organise and develop different kinds of relationships and social interactions^1^,^2^,^3^. From a temporal perspective, the evolution of cooperation is inseparably linked to the socio-ecological context in which resource availability played an important role. In fact, several studies show that the availability and access to resources had a substantial impact on the anatomical and functional development of our species^4^,^5^ and also shaped our strategic behaviour that explains social dynamics^6^,^7^.

In most studies about humankind’s evolution and its historical dynamics, the exploitation of resources recurrently appears as a factor of study, such as human demographic expansions^8^,^9^, the depletion of resources^10^,^11^,^12^, social organisation and social networks^13^,^14^, changes or innovations in social and economic relationships^15^,^16^,^17^, the development of hierarchical and complex societies^18^,^19^,^20^,^21^ and even competition and conflict for resources^22^,^23^. However, an explanatory analysis of how cooperative behaviours (e.g. the sharing of the product of exploitation and access to resources) evolve when dealing with changes in resource availability remains largely unexplored.

In order to provide new insights about the relationship between cooperation and resources, we have developed an agent-based model (ABM)^24^ and used computer simulations to study the conditions that promote cooperation in hunter-gatherer (HG) societies under different resource-stress episodes. The model is a stylised abstraction of the basic mechanisms that explain the emergence of cooperation. It does not consider food storage facilities or techniques that may condition cooperation (see the review in Angourakis *et al*.)^25^,^26^,^27^,^28^,^29^, nor does it introduce other complex assumptions such as social networks or norms. We can claim that the model explores essential questions of classical anthropology regarding the importance of cooperation in human history^30^,^31^,^32^,^33^.

## Background

Cooperation and sharing have been viewed as a hallmark of HG societies^33^,^34^,^35^. These practices have been identified as a long-term strategy to improve the level of wellness necessary for managing risks related to the uneven distribution of resources through time and space^36^,^37^ as well as to increase reputation and enhance social capital in the context of sporadic food surplus^35^,^38^,^39^. Moreover, cooperation has been proposed as a key factor in the expansion of modern humans to the extent that it reduces intraspecific competition and increases population carrying capacities, per-capita growth rates and stability^9^. Several researchers have also highlighted that cooperative practices and sharing may have been limited by the appearance of powerful imbalances between population and resources^28^,^39^. Ethnographic and archaeological studies provide plenty of examples related to the relationship between economic crisis and strategies carried out by HG groups to overcome food shortages (migration, group fission, infanticide, sexual abstention or the abandonment of old or sick people, among others^40^,^41^). Thus, a decrease in resource availability would have promoted cooperation whenever reciprocity was an operational strategy, but once resource scarcity reached a given threshold and reciprocity became less probable, cooperation would have been a useless strategy. The challenge is to identify when this threshold was reached and what options societies could choose from to overcome the new scenario.

In order to accomplish this aim, we developed an agent-based model. Modelling allows us to explore multiple environmental scenarios^42^,^43^ that can address several of these problems. Agent based modelling has been used within the frame of evolutionary game theory in cooperation^44^,^45^,^46^ but also in several archaeological studies (see Lake^47^ for a review of this topic). While there are simulations that analyse the relative contributions of hunting and gathering to the diet^48^, most models focus on how specific tactics relate to overexploitation^49^ or carrying capacities^50^, or on how a given population recovers depending on hunting pressure or overharvesting^51^,^52^,^53^. More recently, the role played by cooperation and store efficiency in the emergence and maintenance of common stocks^25^ and the influence of excessive abundance of common resources in the context of public goods games^54^ have been analysed. However, food storage has not been identified as a spreading strategy in many HG societies and remains a topic that needs further development^55^.

## An Agent-Based Model

The next section describes the model following a compact version of the ODD documentation protocol^56^. The computational model is implemented in NetLogo 5.3^57^ and the corresponding source code can be downloaded at the following website: https://www.openabm.org/model/5287/.

## Overview: Purpose

Cooperation Under Resource Pressure (CURP) is an agent-based model designed to explore the evolution of cooperation under different resource scenarios that shape individual needs for survival. The model is a very stylised abstraction, where resource pressure is modelled by a stochastic process of acquiring resources (i.e. *prob-resource*) and by a parameter of the minimal proportion of the resource unit necessary for survival (i.e. *min-energy*). The model offers different insights about how resource pressure can change the cooperative behaviour of any group of individuals, although we have a particular interest in the evolution of sharing practices in HG societies.

Other assumptions incorporated in the model are tested:

- The size of the population.
- The exploration of individuals, modelled by a probability of mutation parameter.
- The size of the tournaments used to model the sharing process and the imitation process.

## Overview: Entities, State Variables and Scales

The CURP model is an artificial society of N *people* agents that represent individuals. The number of *people* agents in the model remains constant during simulation. The state variables that characterise each agent are defined in Table 1. The *people* agent’s strategy is defined by the values of *given-energy* and *correlation* variables. *People* agents are not embedded in any spatial structure and can interact with each other with equal probability (well-mixed population assumption).

The study parameters of the model (Table 2) are the exogenous variables established by the user that define a simulation scenario, i.e. a computational experiment, and remain constant in each run.

To simplify the model, we assume that resources provide a unit of energy to anyone who finds them. A *people* agent who gets resources attains a unit of energy and shares a proportion of this unit, i.e. *given-energy*, with other unlucky agents. The resources necessary for survival are defined as a proportion of the unit of energy, i.e. *min-energy*. We do not define any temporal scale in the model. Time periods have no meaning and the analysis is focused on the asymptotic behaviour of the model.

## Overview: Process and Scheduling

**Figure 1:**
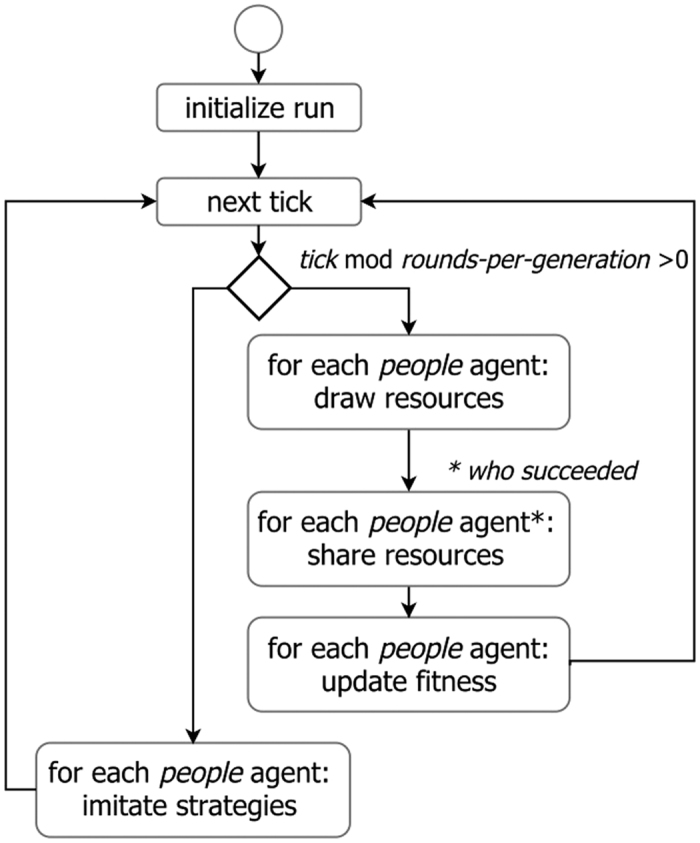
Flow diagram of the schedule of execution. The order in which *people* agents are chosen in “for each” statements is always random to avoid bias in agent selection.

The scheduling is formed by a set of events that take place sequentially in discrete time periods (see Fig. 1). *People* agents perform actions in a random order, avoiding any priority for first-acting consequences. The update of the state variables is asynchronous.

In each time period, each *people* agent draws resources and gets a unit of energy with probability equal to *prob-resource*. Then, each successful *people* agent shares resources in two steps. First, she chooses a donee among a set of unlucky *people* agents. Second, she gives her a *given-energy* proportion of the unit of energy; no donee will receive any more energy from other donors if she gets more energy than the survival threshold defined by *min-energy*.

For each donor, the set of possible donees is defined as a sampling among all agents who did not get resources (by themselves or from other donors), with the set size limited by *sharing-tournament-size*. The selection of a donee from this set is conditioned by the value of the *people* agent’s *correlation*, a variable defined in the interval [−1, 1]. When the *correlation* is positive, a *people* agent chooses the most cooperative individual in the set (that one with the highest *given-energy* value) with a probability equal to *correlation.* Otherwise, she chooses one individual randomly. In the other case, when the *correlation* is negative, a *people* agent chooses with a probability equal to the absolute value of *correlation* the least cooperative individual (the one with the lowest *given-energy* value). Otherwise, she chooses an individual at random.

Finally, each *people* agent updates her fitness, defined as the number of non-starving time periods, increasing a unit if she has more energy than the *min-energy* survival threshold. The process of acquiring and sharing resources and updating fitness is repeated in *rounds-per-generation* time periods. After this time, each *people* agent updates her strategy, i.e. the *given-energy* and *correlation* variables, as follows: first she samples *strategy-tournament-size people* agents of the population, then she imitates the best strategy, i.e. the strategy with the highest *fitness* if the corresponding *fitness* is greater than her own.

Moreover, each *people* agent always randomly chooses a strategy between the strategy space with a *prob-mutation* probability. This assumption responds to the hypothesis that a *people* agent may prefer to explore new strategies, or there may be some errors in the imitation process.

## Initialisation

The user initialises a run by selecting the study parameters’ values in the interface, corresponding to the scenario to be simulated. The *people* agents are then created according to this parameterisation.

## Design Concepts

The basic principle underlying this model is a problem of cooperation. *People* agents face an unknown distribution of resources. Sometimes they succeed, but sometimes they do not. Sharing resources can imply a cost in terms of survival, because the proportion of energy shared by a donor is not conditioned by the *min-energy* survival threshold, i.e. a donor always gives a *given-energy* proportion of her current energy, even though she eventually remains with less energy than the minimum necessary for survival by doing so. On the other hand, living in a population where everyone shares may reduce uncertainty and increase the probability of survival because an individual can get resources by herself or from the generosity of others.

Another interesting question that arises in this problem of cooperation is the decision of who to give resources to. One might expect that some mechanism of reciprocity emerges in these situations; for instance, individuals might prefer to share with others who were generous in the past. Instead of assuming direct reciprocity, the CURP model incorporates a more general rule of selection into the agent’s strategic behaviour. An individual chooses whom to share with considering the level of generosity and depending on the value of *correlation*; a positive value represents a preference for generous individuals (indirect reciprocity), a negative value represents a preference for selfish individuals and a zero value represents indifference.

In the CURP model, the evolution of cooperation is the evolution of the level of generosity represented by the *given-energy* variable and the type of reciprocity represented by the *correlation* variable. The evolution of cooperation is not obvious when the probability distribution of resources and the survival threshold change. Individuals in the population will adapt their strategic behaviour in response to the resource stochasticity and survival pressure in a way that is not easily predictable.

## General Behaviour

The parameter space that defines the experiments is summarised in Table 3. A priori, the main study parameters of the CURP model that determine the resource pressure are *prob-resource* and *min-energy*. However, there are other parameters primarily assumed to make the model run that may influence the model dynamics significantly. To quantify the importance of the study parameters, we applied a test of variable importance based on Random Forests using Latin Hypercube Sampling (LHS)^58^. LHS divides the range of each parameter (see Table 3) into N = 3000 strata of equal probability and generates N experiments using a random sampling of these strata, ensuring that each stratum is presented in one experiment. This statistical technique is very useful for sampling multidimensional distributions.

Each LHS experiment was run 5 × 10^3^ generations, a set of time periods sufficient for reaching the stationary regime. For all simulations, the average of the strategic variables *given-energy* and *correlation* in the population was recorded.

The test of variable importance is based on Random Forests (RFs)^59^. RFs are a statistical learning technique used in many classification and regression problems^60^. An RF combines predictions of a set of trees, using a bootstrapping sampling of the dataset to fit each tree. In our case, we are not interested in RFs’ predictive capabilities, but in identifying the most important variables^61^. The sampling technique used in RFs always leaves an unused subset of data called “Out-Of Bag” (OOB). For each OOB set, each variable is permuted at random and then computed as the Mean Standard Error (MSE) of the RF. Finally, the importance of a variable corresponds to the increase in MSE after permutation.

To simplify the underlying regression problem, we applied PCA to convert the set of state variables, i.e. *given-energy, correlation* along with the time period variable, into principal components. Then, we used the first component as the dependent variable in a RF. These algorithms typically include two free parameters that are not directly learnt within the data: (i) the number of trees (N_tress_) and (ii) the cardinal of the random subset of variables (Max_features_) that are considered as candidates in each split of the trees (random subspace method)^62^. The optimal RF parameters are determined using 10-fold cross-validation^63^,^64^ in a grid search process. This method involves the partition of the data in 10 equal subsamples, and using 9 for training and 1 for the test. The process is repeated for each possible parameter combination considered rotating the subsamples used for training and testing, and the results are averaged. In particular, we have explored N_trees_ = {100, 200, 300, 400, 500, 600, 800, 1000} and Max_features_ = {2, 3, 4, 5, 6, 7}. The optimal parameters found through this process (N_trees_ = 800, Max_features_ = 5) are used to train a RF with all the available data and to perform the variable importance analysis. Table 4 shows a ranking of the relative importance of the study parameters.

The variable importance analysis shows that the general model behaviour depends mainly on two study parameters, *prob-resource* and *min-energy*, which define the resource pressure. Considering this, we performed a more detailed study of both variables to understand the system dynamics.

## The Effects of Resource Pressure

Now we define a set of experiments that correspond to different scenarios of resource pressure and study the emergence of cooperation via simulation. The two *prob-resource* and *min-energy* study parameters have been evenly sampled over the range [0.2, 0.8] in steps of 0.1. The rest of the parameterisation was arbitrarily set to: n-people = 300, rounds-per-generation = 10, prob-mutation = 0.01, sharing-tournament-size = 0.01, strategy-tournament-size = 0.01. Each experiment was run 5 × 10^3^ generations and replicated 30 times.

The resource pressure in the CURP model has two dimensions: a stochastic dimension determined by the probability of acquiring resources (i.e. *prob-resource*) and a quantitative dimension determined by the minimal proportion of the resource unit necessary for survival (i.e. *min-energy*). The resource pressure can be interpreted in terms of the specific conjuncture regarding availability of resources that individuals face. The combination of both parameters in the interval of values sampled sketches a broad series of scenarios, ranging from a very low-stress scenario when *prob-resource* is 0.8 and *min-energy* is 0.2, to a very high-stress scenario when *prob-resource* is 0.2 and *min-energy* is 0.8, and intermediate levels of stress for the rest.

Our analysis of the results focuses on the asymptotic behaviour of the population. The strategic behaviour of the agents is analysed in the state space defined by the strategic variables *given-energy* and *correlation*. To facilitate this analysis, we estimated the probability density function using a multivariate kernel density estimation^65^. The data used to estimate the density function are the state of the system during the simulations from the 10,000 to 50,000 time periods, removing the first 10,000 periods to reduce the transient effect of the initial conditions.

**Figure 2:**
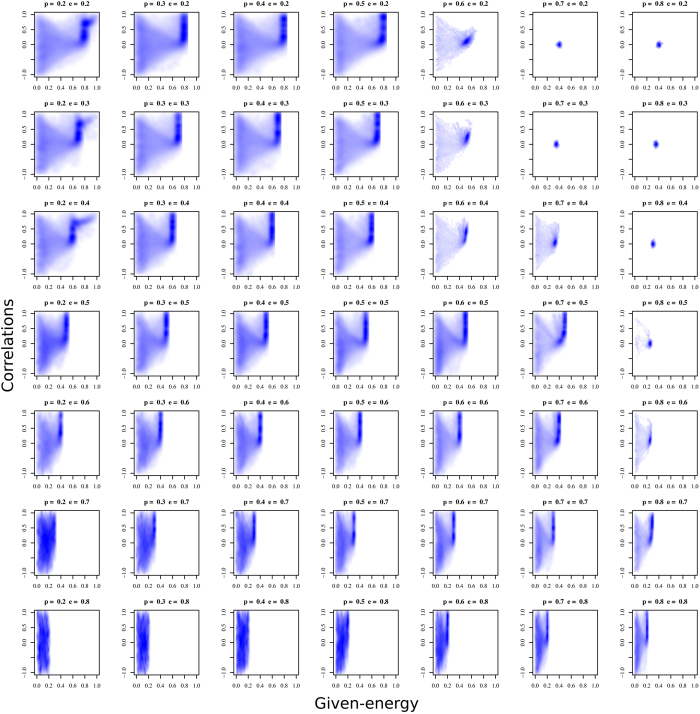
Matrix of plots of the probability density function of the state space for different stress scenarios. Each subfigure represents the density of the simulation outputs of the model in the space given-energy (horizontal axis) and correlation (vertical axis) for different values of *prob-resource (p* in titles) and *min-energy (e* in titles) parameters. In this smoothed color density scatterplot, darker values of (blue) color imply higher probability of a simulation of finish with these output values. The *prob-resource* grows from left to right, and the *min-energy* from top to bottom.

Figure 2 shows the results as a matrix of plots. Each plot grows in *prob-resource* from left to right, and grows in *min-energy* from top to bottom. Left plots show a low probability of finding resources, while right plots indicate a high probability, hence the system is more stressed in terms of the stochasticity when we go towards the left in Fig. 2. On the other hand, the top row of plots in the figure represents low values of the energy threshold needed for survival, which increases towards the bottom. As a result, the system is stressed regarding the quantity when we go towards the bottom of the figure.

Taking the results of Fig. 2 into account, we can define three different regimes based on the values of the parameter combinations.

First, there is **a low-stress regime** (i.e. low values of *min-energy* and high values of *prob-resource*) in the top-right area of the plot matrix. Survival in this situation is very likely. Any individual strategy obtains high fitness and therefore the selection mechanism barely takes action. Movements in the state space are given to random drift, but since selection is not important and the strategies do not favour any specific direction, the average strategic behaviour of the population remains at almost constant values.

Second, there is **a high-stress regime** (i.e. high values of *min-energy* and low values of *prob-resource*) in the bottom-left area. Dynamics are characterised by very unstable behaviour. The high threshold of *min-energy* and the scarcity of resources due to low *prob-resource* favour low *given-energy* strategies. However, the population simultaneously happens to be constantly searching for strategies to ensure survival. The population is pulled towards any random successful strategy as a consequence of the selection pressure, but any situation is satisfactory enough and the population continuously evolves in the quest for survival.

Finally, there is **a third**, **intermediate-stress** or moderate survival stress regime running diagonal to the plot matrix, from left to right and top to bottom. In all these situations, population coordinates increase the probabilities of survival and reduce the risk of remaining under the threshold in an evolutionary way. Strategies are characterised by retaining the resources strictly necessary for survival and transferring the rest to the population, but in a structured manner, as strategies of positive correlation (preferential giving to those that have transferred more resources in previous situations) are evolutionarily favoured. Populations self-organise in an **indirect reciprocity system** in which the norm is to share the part of the resource that is not strictly necessary for survival in a certain time period, hoping that the rest of the population will do the same in the future. Therefore, the probability of being under the threshold is collectively reduced. The marginal benefit of keeping the surplus in the model is considered as a second-order objective compared with reaching the survival threshold in each time period. In moderate-stress regimes, the evolutionary pressure causes this benefit to be renounced by the individuals to increase the common probability of survival of the rest of the population.

## Discussion

The results offered by the CURP model have interesting consequences for analysing human social behaviour related to cooperation. As explained in the first few pages, cooperation is a paramount element for understanding human social relationships and the development of sociality. HG societies may implement a wide variety of strategies that can change according to different elements such as the general socio-ecological context, in which different population densities deal with distinct distributions and concentrations of resources, socio-historical dynamics and internal social changes. CURP parametrises both the probability of finding resources and the need for a specific amount of energy for survival, reproducing what might be the different socio-ecological settings that HG societies faced throughout human evolution and world colonisation. Although technology and production have been traditionally argued as the main factors that explain human capabilities to face new landscapes or changing resource availability, the results suggest that a modification of distribution and consumption patterns allow HG societies to deal with changing resource availability.

Within the identified regimes in CURP, the emergence of cooperative behaviours is the most relevant trend, showing that cooperation is a strategy that depends on the possibility of survival, but also on the expectancy of reciprocity. These results also reflect that communal behaviour varies along this continuum; despite being stable in low- and intermediate-stress regimes, it is unstable in high-stress regimes. While stability provides the possibility of reproducing prevailing dynamics and testing its effect through generations (meaning that cooperation is always a communal strategy even though it is performed individually), unstable strategies are usually individual.

Low-stress scenarios correspond to contexts in which no environmental or social elements push towards the selection of a particular strategy, so that agents behave indistinctly according to previous behaviour. Besides this, and because of the absence of forces that drive the development of specific strategies, these contexts typically promote only slow transitions at the population level. Given the abundance of resources, all strategies are successful and cooperation does not increase or decrease.

The high-stress scenario could be tagged as a context of crisis and corresponds to contexts where resources become scarcer, surpluses disappear and individual needs are not always fulfilled. These conditions transform previous organisational strategies, which are no longer considered efficient anymore. Social organisational innovation may be the key to resolving the critical context (death by starvation and the consequent population reduction is not considered in the parameters of the model). While the promotion of cooperation reduces intraspecific competition^9^, such high-stress contexts would promote strategic and organisational change^66^,^67^. In this case, this means that cooperation and distribution are modified and social agents try alternatives to prevailing behaviour, even though none of them stands out and becomes predominant. The model’s absence of an operative way out of the crisis builds a context where the most important trait is a constant quest for solutions: cooperation and other innovative options emerge in this context, even though none of them are stabilising. Consequently, any specific strategy becomes prevalent, but is quickly substituted by another when adopted by a majority. Population-resource imbalance has very often been considered one of the elements that push societies towards organisational changes^18^,^68^, which may increase complexity^69^. Current results from the CURP model strengthen the idea that such changes promote both innovation and social transitions.

Finally, the intermediate-stress context is the most interesting and important in CURP. In this scenario, resources appear unevenly distributed and energy requirements also change, either by environmental variations or by social changes. Crisis scenarios appear as sporadic episodes showing different intensities. This modelled context is a robust example of an emergent mechanism of social cooperation: this strategy successfully establishes its ability to improve the survival conditions of HG societies.

In this case, the heterogeneous accessibility to resources promotes the development of their specific redistribution once they are within the social domain through different types of sharing behaviours. Once individual requirements for survival are reached, surpluses (which are useless, because the model does not consider storage facilities or technologies) are given within a common strategy that prioritises group survival.

This environmental variability, considered within a specific territory, or the progressive expansion of *Homo sapiens* throughout the planet, was the frame for stabilising cooperative strategies aimed at minimising the effects of this variability (e.g. subsistence instability) through sharing behaviours. In the absence of delayed consumption provided by storage, group consumption is promoted by sharing and reciprocity in most HG societies to deal with uncertainty^70^,^71^,^72^,^73^. The origins of *Homo sapiens* took place in equatorial areas, but the occupation of other territories, in the highest latitudes, established new contexts with different distribution of resources. In these contexts, not only technological innovation but also flexible organisation, and consequently innovation, may have been important factors for survival^74^. Therefore, the cooperative enterprise that we find in many small-scale societies may be the result of a long-run growing strategy to fluctuating resources^75^ and may be considered one of the most important social tools in the success of the human species^9^.

The existence of social norms in small-scale societies promoting sharing demonstrates the importance of this strategy for the sustenance of the group^76^. Several pieces of ethnographic evidence point to sharing as a particular form of cooperation that helps to eliminate the risk of shortages^77^,^78^,^79^,^80^ and is promoted during times of scarcity (see the Pumé from Venezuela^81^, the Copper Inuit^82^ and others). In addition, food sharing provides the material basis for other cooperative behaviours such as the establishment of marriages or political alliances^38^,^83^,^84^,^85^. It has also been argued that sharing accomplishes a signal function that reinforces the set of cooperative behaviours that accompany it^86^,^87^.

Social identity, common knowledge and their transmission are key elements of this mechanism: the existence of the social memory of critical periods consolidates the cooperative mechanisms^88^. As a result, the existence of cooperative mechanisms, with their successful options, consolidates the social bonds of HG groups, aiming to establish social identities^6^,^86^,^89^,^90^.

## Conclusions

Variations in resource availability have been used extensively as explanatory causes for different social changes throughout the course of human history. Although organisational changes are considered part of these explanations, research on the emergence of cooperation and the development of common strategies within these processes has not yet received much attention. Notwithstanding, the human ability to develop and adopt cooperative strategies is precisely the element that has given such macro-ecological advantages to humankind, promoting survival in different contexts. This paper proposes a model for understanding the influence of resource availability on cooperation. The results show that resource pressure—the balance between population and resources—affects cooperative behaviours, specifically sharing practices.

The CURP model illustrates how societies adapt to fluctuating conditions through organisational changes. It shows that the discontinued intermediate availability of resources favours mixed cooperative strategies in which the population prioritises the individual use of the resource to guarantee survival, but sharing practices emerge as cooperative strategies to increase population carrying capacities and stability at the same time. The model does not include any mechanism to efficiently store and preserve food resources, leading to strategies in which the surplus may be exchanged as a risk reduction policy during subsequent periods that are not as fruitful. In this context, the model suggests that reputation and generosity could emerge as social capital mechanisms, which implies cultural and social. In high-pressure situations, societies dynamically explore and test new survival mechanisms: exploration is a strong trait in this scenario. The volatility of survival strategies pushes the population to highly diverse but unstable situations, inducing a very active dynamic. Hunter-gatherer groups may absorb these processes by reducing the population (death, migration, group-splitting strategies, etc.) or by increasing resource availability, like by developing social and/or technological innovation, for instance. On the contrary, in low-stress situations, social norms and sharing practices are not exposed to adaptive pressure; consequently, the co-existence of different dynamic practices may emerge. Social change is produced by drift, so transitions are not as abrupt as in the other two scenarios. In fact, initial homogeneous norms could remain in societies over long time periods. In this context, the historical dependence of previous norms may be very relevant.

As a concluding remark, our results indicate that the existence of different management strategies may be related to specific conjunctures, but also to historical dynamics. This is relevant in relation to traditional ideas about HG groups that claim that these societies lack their own history and evolution and that they do not innovate or change according to specific conjunctures.

## Additional Information

**How to cite this article:** Pereda, M. *et al*. Emergence and Evolution of Cooperation Under Resource Pressure. *Sci. Rep.* **7**, 45574; doi: 10.1038/srep45574 (2017).

**Publisher's note:** Springer Nature remains neutral with regard to jurisdictional claims in published maps and institutional affiliations.

## Figures and Tables

**Table 1:** *People* agent’s state variables.

Parameter name	Brief description
*given-energy*	The proportion of the resource unit that a *people* agent is willing to share.
*correlation*	This ranges from −1 to 1 and determines the probability of choosing a donee as follows: for positive values, it represents the probability of selecting the most cooperative donee (with the highest given-energy) between the set of possible donees; for negative values, its absolute value represents the probability of selecting the least cooperative donee (with the lowest given-energy) between the sets of possible donees. Otherwise, the donee is chosen randomly.
*fitness*	Fitness is computed as the number of time periods in which the energy obtained by an agent was greater than *min-energy.*

**Table 2:** Study parameters.

Parameter name	Brief description
*n-people*	Number of *people* agents.
*prob-resource*	The probability that a *people* agent gets a unit of resource at each time period.
*min-energy*	The minimal proportion of the resource unit necessary for survival.
*sharing-tournament-size*	The percentage of *people* agents with no resource at a time period that can be chosen as a donee by a particular donor.
*strategy-tournament-size*	The percentage of *people* agents of the population that a particular agent considers in the imitation process.
*prob-mutation*	The probability that a *people* agent decides to follow a new strategy randomly chosen from the strategy space.
*rounds-per-generation*	*People* agents can change their strategy, i.e. *given-energy* and *correlation*, every *rounds-per-generation* time periods.

**Table 3:** Parameter space for LHS analysis.

Parameter name	Range explored
*n-people*	[100,500]
*prob-resource*	[0,1]
*min-energy*	[0,1]
*sharing-tournament-size*	[0,1]
*strategy-tournament-size*	[0.01,1]
*prob-mutation*	[0.01,1]
*rounds-per-generation*	[10,50]

**Table 4:** Ranking of the relative importance of the study parameters.

Parameter name	Relative importance
*prob-resource*	0.3696
*min-energy*	0.2486
*prob-mutation*	0.0923
*strategy-tournament-size*	0.0803
*sharing-tournament-size*	0.0747
*n-people*	0.0732
*rounds-per-generation*	0.0613

The scikit-learn python library^91^ has been used to fit an RF with the next parametrisation: N_trees_ = 800, Max_features_ = 5, depth = 1000. The impurity node has been used as the measure of the quality of splits. The importance values are positive and add up to 1.0. The higher the value, the more important the contribution of the corresponding study parameter to the prediction of the RF.
